# Tracking the Impact of COVID-19 and Lockdown Policies on Public Mental Health Using Social Media: Infoveillance Study

**DOI:** 10.2196/39676

**Published:** 2022-10-13

**Authors:** Minghui Li, Yining Hua, Yanhui Liao, Li Zhou, Xue Li, Ling Wang, Jie Yang

**Affiliations:** 1 Department of Big Data in Health Science School of Public Health, Center of Clinical Big Data and Analytics of The Second Affiliated Hospital Zhejiang University School of Medicine Hangzhou China; 2 The Key Laboratory of Intelligent Preventive Medicine of Zhejiang Province Hangzhou China; 3 Department of Biomedical Informatics Harvard Medical School Boston, MA United States; 4 Division of General Internal Medicine and Primary Care Department of Medicine Brigham and Women’s Hospital Boston, MA United States; 5 Department of Psychiatry, Sir Run Run Shaw Hospital School of Medicine Zhejiang University Hangzhou China; 6 Florence Nightingale Faculty of Nursing, Midwifery & Palliative Care King’s College London London United Kingdom

**Keywords:** COVID-19, mental health, social media, Twitter, topic model, health care workers

## Abstract

**Background:**

The COVID-19 pandemic and its corresponding preventive and control measures have increased the mental burden on the public. Understanding and tracking changes in public mental status can facilitate optimizing public mental health intervention and control strategies.

**Objective:**

This study aimed to build a social media–based pipeline that tracks public mental changes and use it to understand public mental health status regarding the pandemic.

**Methods:**

This study used COVID-19–related tweets posted from February 2020 to April 2022. The tweets were downloaded using unique identifiers through the Twitter application programming interface. We created a lexicon of 4 mental health problems (depression, anxiety, insomnia, and addiction) to identify mental health–related tweets and developed a dictionary for identifying health care workers. We analyzed temporal and geographic distributions of public mental health status during the pandemic and further compared distributions among health care workers versus the general public, supplemented by topic modeling on their underlying foci. Finally, we used interrupted time series analysis to examine the statewide impact of a lockdown policy on public mental health in 12 states.

**Results:**

We extracted 4,213,005 tweets related to mental health and COVID-19 from 2,316,817 users. Of these tweets, 2,161,357 (51.3%) were related to “depression,” whereas 1,923,635 (45.66%), 225,205 (5.35%), and 150,006 (3.56%) were related to “anxiety,” “insomnia,” and “addiction,” respectively. Compared to the general public, health care workers had higher risks of all 4 types of problems (all *P*<.001), and they were more concerned about clinical topics than everyday issues (eg, “students’ pressure,” “panic buying,” and “fuel problems”) than the general public. Finally, the lockdown policy had significant associations with public mental health in 4 out of the 12 states we studied, among which Pennsylvania showed a positive association, whereas Michigan, North Carolina, and Ohio showed the opposite (all *P*<.05).

**Conclusions:**

The impact of COVID-19 and the corresponding control measures on the public’s mental status is dynamic and shows variability among different cohorts regarding disease types, occupations, and regional groups. Health agencies and policy makers should primarily focus on depression (reported by 51.3% of the tweets) and insomnia (which has had an ever-increasing trend since the beginning of the pandemic), especially among health care workers. Our pipeline timely tracks and analyzes public mental health changes, especially when primary studies and large-scale surveys are difficult to conduct.

## Introduction

The global COVID-19 pandemic has drastically changed people’s daily lives since the first confirmed case in December 2019 [1]. It has led to high hospitalization and fatality and negatively impacted public mental health [2,3]. Mental health problems cover a wide range of populations during the pandemic. The causes include but are not limited to the infection and death of relatives and friends, fear of illness, isolation brought by quarantine [4,5], and stress from unemployment [6]. At the same time, specific subpopulations such as children and adolescents [7,8], students [9,10], patients with COVID-19 [11], and health care workers [12,13] are particularly vulnerable to psychological disorders during the pandemic.

Studies have pointed out that health care workers in the United States experience psychological distress, facing high levels of anxiety, depression, and burnout during the pandemic [14]. The underlying reasons could be higher exposure risks to the virus and overwhelming workload [15,16]. Although there is literature on studying the mental health status of health care workers during the pandemic period, existing research primarily focuses on retrospective cross-sectional studies [13,14,16-19]. Therefore, it is necessary to study the dynamic characteristics of their mental status, identify general concerns, and provide timely support [20,21].

Due to their large scale, immediacy, and comprehensive coverage, social media platforms (such as Twitter, Facebook, and Weibo) have been vital data sources of research to analyze public perceptions timely when primary studies and large-scale surveys are difficult to be conducted. For example, Chew et al [22] used Twitter to study misinformation during the 2009 H1N1 pandemic, and Masri et al [23] found that new case trends can be predicted 1 week ahead based on related tweets for the 2015 Zika epidemic. Similarly, numerous studies have used social media to monitor public perceptions on topics such as enforced remote work [24], vaccines [25,26], drug use [27], mask wearing [28], and so on. Meanwhile, Berry et al [29] pointed out through a study with both quantitative and qualitative approaches that people are willing to discuss mental health problems on Twitter for varied reasons, including the sense of community and Twitter being a safe space for expression, coping, empowerment, etc. However, existing literature on public mental health during the pandemic using Twitter data [30-33] either has short study periods and small sample sizes or does not focus on subtypes of mental health problems and subgroup prevalence. More granular study designs and more comprehensive data are needed for such studies.

Finally, there is inconsistency in studying the effect of lockdown policies—one of the most highly debated topics related to mental health during the pandemic. Das et al [34] found that “state lockdown policies precede greater mental health symptoms.” In contrast, Adams-Prassl et al [35] found that “the lockdown measures lowered mental health by 0.083 standard deviations.”

To fill in these research gaps and potentially resolve the inconsistency, this study aimed to use related data from February 1, 2020—the beginning of the pandemic—to April 30, 2022, to analyze public mental status, problem types, their temporal and geographic distributions during COVID-19, as well as the effects of lockdown policies on public mental health across states (Figure S1 in Multimedia Appendix 1). In detail, we used this study to answer the 4 following research questions:

1. What types of mental health problems were the most frequent?
2. What mental health–related topics were the public the most concerned about, and how did relevant discussions change over time?
3. Are there differences in mental health concerns between the general population and health care workers?
4. How did lockdown policies impact public mental health?

To answer question 1, two mental health experts from our teams curated a mental health lexicon for Twitter that categorizes related tweets into 4 common mental health problems: anxiety, depression, insomnia, and addiction. Based on this lexicon, we extracted related tweets and visualized their distributions by week and state. To answer questions 2 and 3, we built a pipeline to identify potential health care workers, used a topic model to summarize related tweets into 16 topics, and compared the topic distributions among health care workers and the general population. To answer question 4, we identified tweets related to mental issues and compared their proportions before and after lockdown policies across different US states.

## Methods

### Data Collection

We collected and downloaded COVID-19–related tweets from February 1, 2020, to April 30, 2022, from Twitter’s application programming interface using the unique tweet ID provided by an open-source COVID-19 tweet database [36]. The downloaded data contained full tweet texts and the corresponding metadata, including created time, user information, tweet status, etc. We further filtered out non–English-language and retweeted tweets and kept 471,371,477 tweets. Our data collection process strictly followed Twitter’s privacy and data use management. This study followed the Strengthening the Reporting of Observational Studies in Epidemiology reporting guidelines.

### Ethics Approval

This study was conducted with approval by the Institutional Review Board of Zhejiang University (ZGL202201-2).

### Data Preprocessing and Filtering

**Figure 1:**
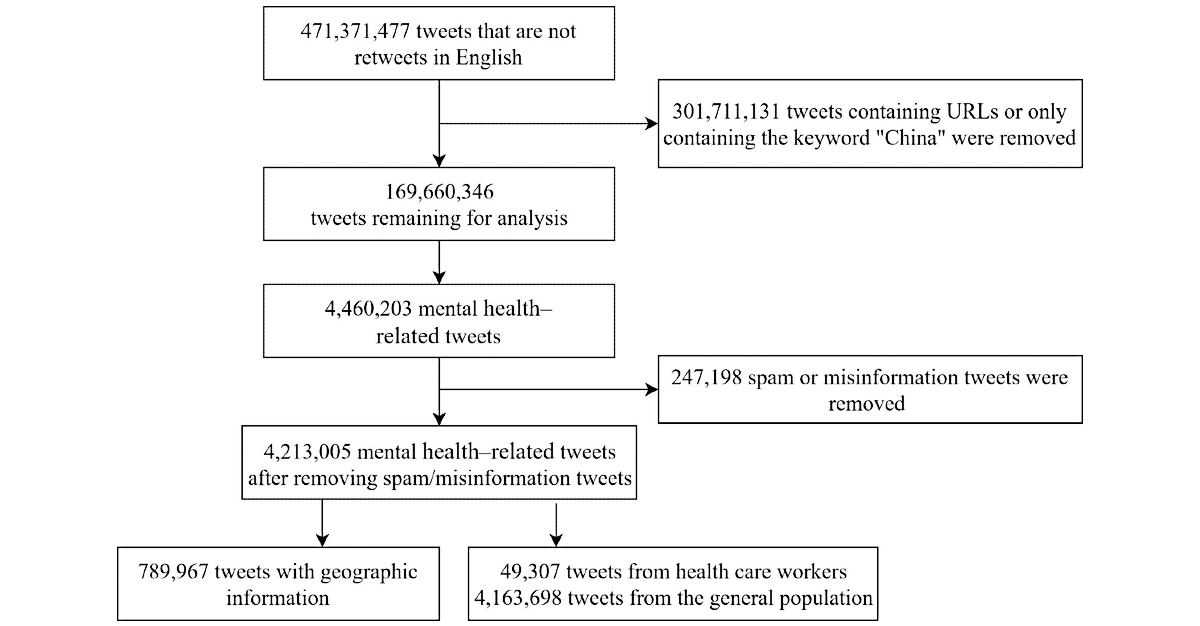
Data collection and preprocessing.

We removed tweets that contain URLs because such tweets often only included summaries or quotations of the original contents (169,660,346 tweets remained). A psychiatrist and a psychologist curated a mental health lexicon with 231 keywords. The keywords were categorized into 4 subgroups: anxiety, depression, insomnia, and addiction (Table S1 in Multimedia Appendix 1). We used this lexicon to extract mental health–related tweets through keyword matching against the preprocessed tweets and identified 4,460,203 tweets. To reduce the impact of spam and misinformation tweets, we removed data from users who posted more than 1000 mental health–related tweets during the study period. The final data set contained 4,213,005 tweets. Figure 1 shows an overview of the data preprocessing process.

### Geographic Information Extraction

The geographic information of users was collected from 2 fields of the tweets: (1) the “place” field in tweet metadata and (2) the “location” variable nested in the “user” field of tweet metadata. The “place” information was chosen as the primary evidence of the users’ geographic information, since it is generated from GPS data and is, therefore, more accurate than the information from the self-reported “location” field. We used a list of US state names to extract users’ geographic information (“Methods” in Multimedia Appendix 1 [37-39]). Tweets from users associated with more than 1 state were removed in this step.

### Topic Model Analysis

The Latent Dirichlet Allocation model [39] was used to conclude the main topics of mental health–related tweets. To create the corpora for topic modeling, we removed all stop words [40] as well as numbers and symbols. The topic model was implemented using the *LdaModel* function of the *Genism* package [40]. We selected the number of topics—a model hyperparameter—based on perplexity and topic coherence (“Methods” in Multimedia Appendix 1 [37-39]).

### Health Care Worker Identification

To identify health care workers, we built a health care worker identification lexicon, whose keywords can be roughly divided into 3 groups: occupation, degree, and the title of the association (“Methods” in Multimedia Appendix 1 [37-39]). The dictionary contained 47 keywords, such as “doctor,” “MD,” “Doctor of Medicine,” “FACP,” etc (Table S2 in Multimedia Appendix 1). We used this lexicon to filter the user’s description and extracted 49,307 tweets from health care workers.

### Statistical Analysis

We applied standard descriptive statistics to summarize the 4 types of mental health–related tweets proportion, including median and IQRs. Wilcoxon matched-pairs signed-ranks test was used to compare differences between health care workers and the general population. Interrupted time series analysis [41] was applied to analyze the lockdown policy’s effects on public mental health (see detailed information in “Methods” in Multimedia Appendix 1 [37-39]). We used Python software (version 3.8) to conduct the statistical analyses and chose a *P* value of .05 as the statistically significant threshold.

## Results

### Collected Data Set

Data preprocessing selected 4,213,005 mental health–related tweets from 2,316,817 users (Figure 1). Among these tweets, 51.3% (2,161,357) were in the “depression” group, 45.66% (n=1,923,635) tweets were in the “anxiety” group, 5.35% (n=225,205) tweets were in the “insomnia” group, and 3.56% (n=150,006) tweets were in the “addiction” group. The sum of the 4 proportions was larger than 100% because some tweets included multiple keywords that belong to different mental health subgroups. Additionally, 789,967 (18.75%) tweets were extracted with their geographic information, and health care workers posted 49,307 (1.17%) tweets (from 21,963 users).

### Temporal Distribution of Mental Health–Related Tweets

**Figure 2:**
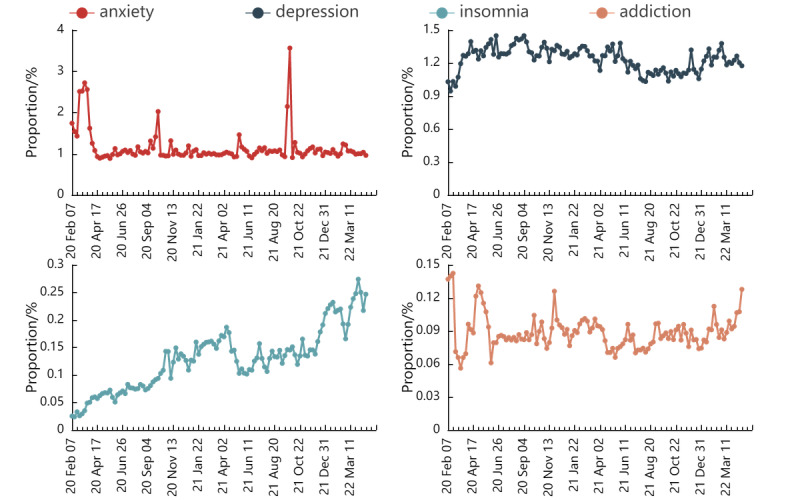
Trends of 4 types of mental health symptom–related tweets by the proportion of tweets.

The trends of the weekly numbers of COVID-19 new cases and mental health–related tweets in 4 subgroups are shown in Figure S2 in Multimedia Appendix 1. The number of tweets of mental health problems reached their first peak from February 29 to April 4, 2020. We calculated and visualized the proportions of mental health–related tweets among all COVID-19–related tweets in Figure 2. The proportion curve of anxiety-related tweets had 3 dominant peaks in March 2020, October 2020, and September 2021. The curve of insomnia-related tweets continually increased during the study period, whereas no specific trends were observed in the curves of depression and addiction.

### Geographic Distribution of Mental Health–Related Tweets in the United States

**Figure 3:**
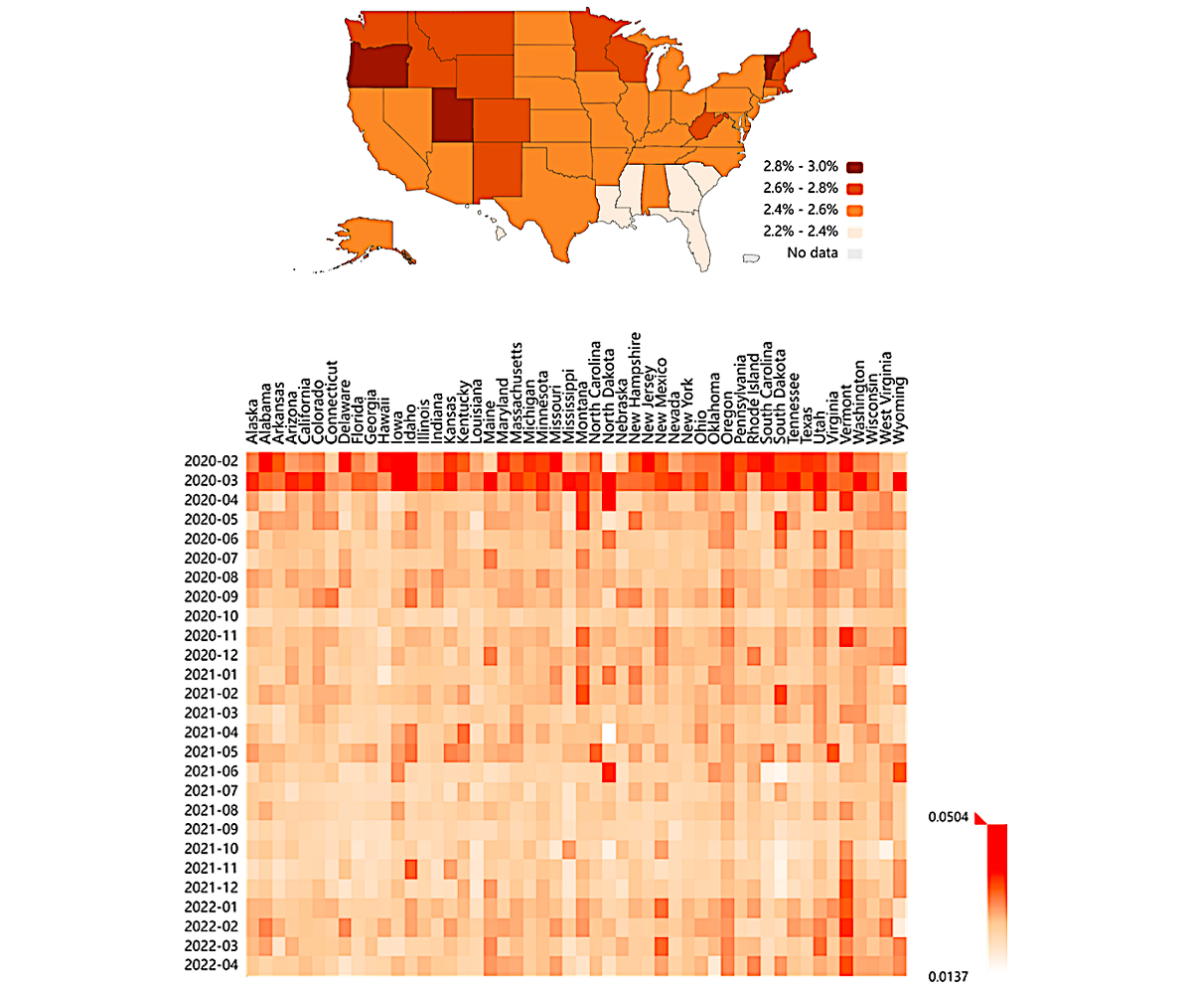
Proportion distribution of mental health–related tweets in the United States.

Figure 3 shows the proportion of mental health–related tweets among all COVID-19–related tweets in each US state from February 1, 2020, to April 30, 2022, and visualizes the monthly tweet proportion for all the 50 US states (concrete proportions and 95% CIs are listed in Multimedia Appendix 2). Vermont, Oregon, and Utah were the 3 states with the highest proportions of mental health–related tweets, whereas Mississippi, Hawaii, and Louisiana had the lowest proportions. The first 2 months had a more substantial proportion of mental health–related tweets than the following months across most states.

### Topics of Mental Health–Related Tweets

**Figure 4:**
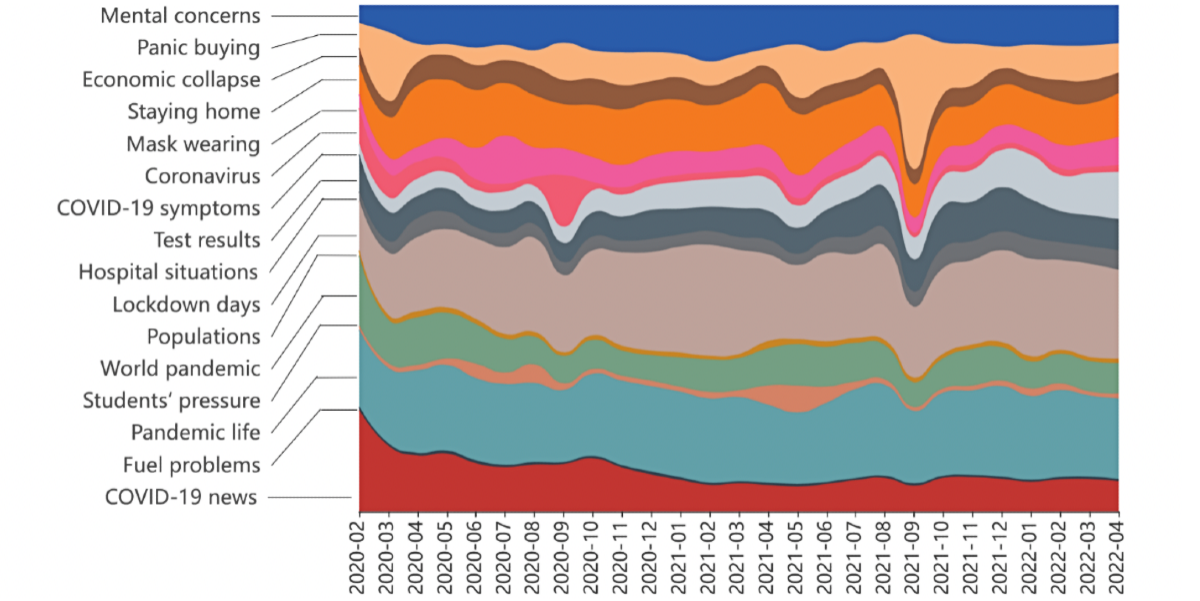
Dynamic characteristics of topic proportions.

The most frequent terms for mental health–related tweets were “people,” “worried,” “shame,” “panic,” “lockdown,” “anxiety,” “mask,” etc (Figure S3 in Multimedia Appendix 1). We chose 16 to be the number of topics based on the perplexity and coherence (“Methods” and Figure S4 in Multimedia Appendix 1 [37-39]). Topics and the corresponding top 20 most probable unigrams and bigrams are displayed in Table S3 in Multimedia Appendix 1. We assigned each topic with a topic name based on the keywords. For example, a topic having the keywords “college,” “student,” “stress,” and “exam” indicates that tweets on this topic was likely to have been focused on “students’ pressure.” Except for the issues related to COVID-19 itself, such as “COVID-19 news,” “test results,” and “mask wearing,” the public also showed particular interest in topics such as “economic collapse,” “panic buying,” and “fuel problems.” The 16 topics were then categorized into 6 topic groups: “COVID-19 pandemic,” “preventive measures,” “economic,” “people,” “education,” and “mental health.” Figure 4 shows the dynamic distributions of the investigated topics in relative tweet proportions. The topic “lockdown days” occupied a dominant position during the pandemic most of the time. “COVID-19 news” was frequently mentioned at the beginning of the pandemic but returned to an average level after June 2020. The topic of “panic buying” notably fluctuated in the research period and was relatively large from February to March 2020 and from August to October 2021.

### Mental Health of Health Care Workers

**Figure 5:**
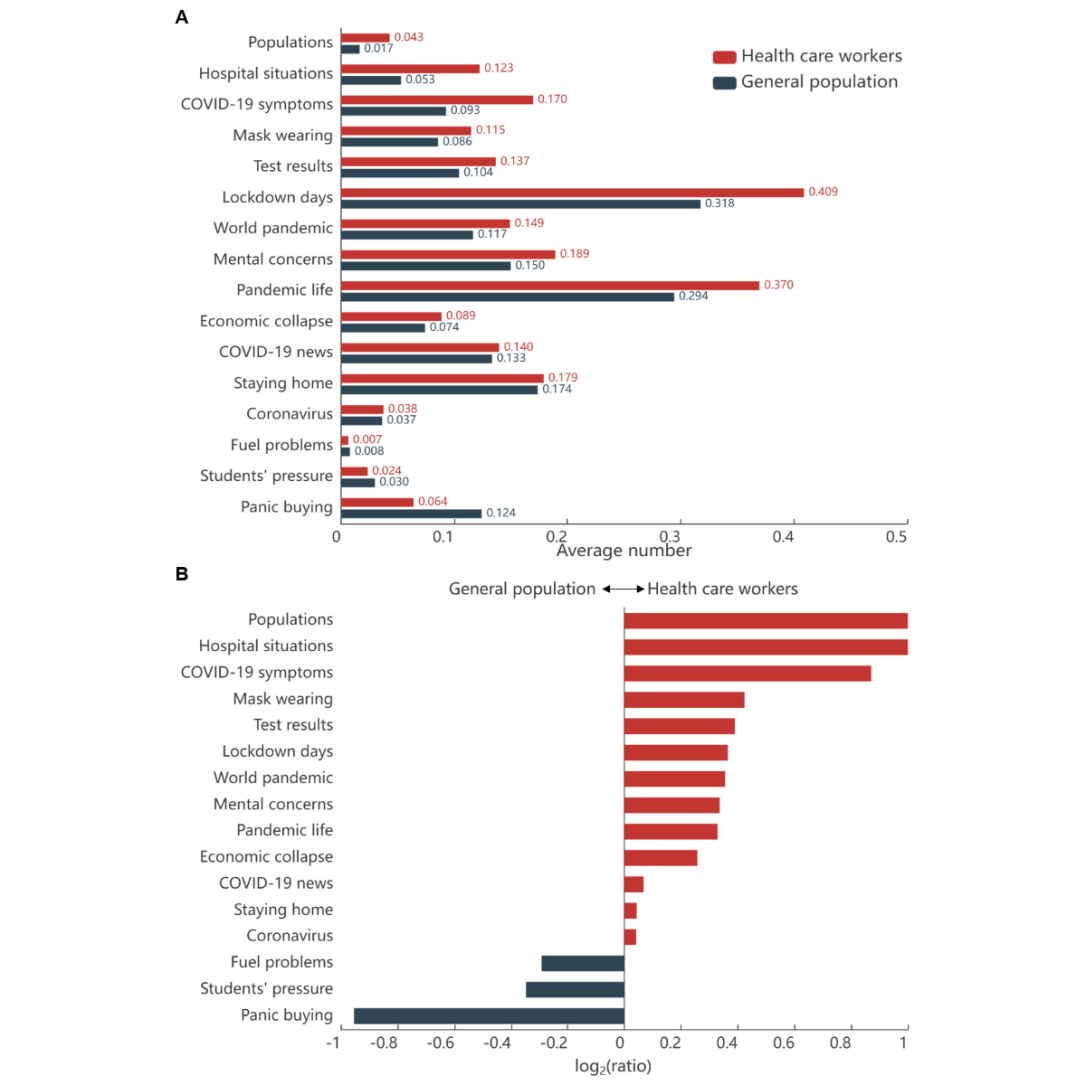
The distribution of tweets in topics for health care workers and the general population. (A) Average number of tweets per user in each topic. (B) Logarithmic ratio of the average number of tweets between health care workers and the general population on each topic. The ratio equals the average number of tweets per user among health care workers divided by the average number of tweets among the general population.

We assessed the differences in the proportions of 4 mental health symptom–related tweets between health care workers and the general population and showed the results in Table 1. Statistical results showed that the proportions of anxiety-, depression-, insomnia-, and addiction-related tweets were significantly higher in health care workers than in the general public (all *P*<.001). Figure 5A shows the average number of tweets per user on different topics. “Lockdown days” is the top topic discussed by both health care workers and the general population. To visualize the difference in topic distribution between health care workers and the general population, we visualized the ratios of the average number of tweets by topic for the 2 groups in Figure 5B. It demonstrates that health care workers discussed more on 13 topics, especially clinical-related topics such as “hospital situations,” “COVID-19 symptoms,” and “mask wearing.” Conversely, the general population focused on topics such as “fuel problems,” “students’ pressure,” and “panic buying.”

**Table 1:** Comparison of proportions of mental health–related tweets between health care workers and the general population.

Mental health symptom	Health care workers (% tweets), median (IQR^a^)	General population (% tweets), median (IQR^a^)	W	*P* value
Anxiety	1.103 (1.02-1.187)	1.025 (0.956-1.094)	2120	<.001
Depression	1.519 (1.396-1.642)	1.255 (1.171-1.339)	26	<.001
Insomnia	0.251 (0.175-0.328)	0.131 (0.093-0.17)	7	<.001
Addiction	0.139 (0.114-0.164)	0.086 (0.079-0.094)	185	<.001

^a^IQR and Wilcoxon matched-pairs signed-ranks test were applied to compare the differences between the 2 groups.

### Impacts of Lockdown Policies

**Figure 6:**
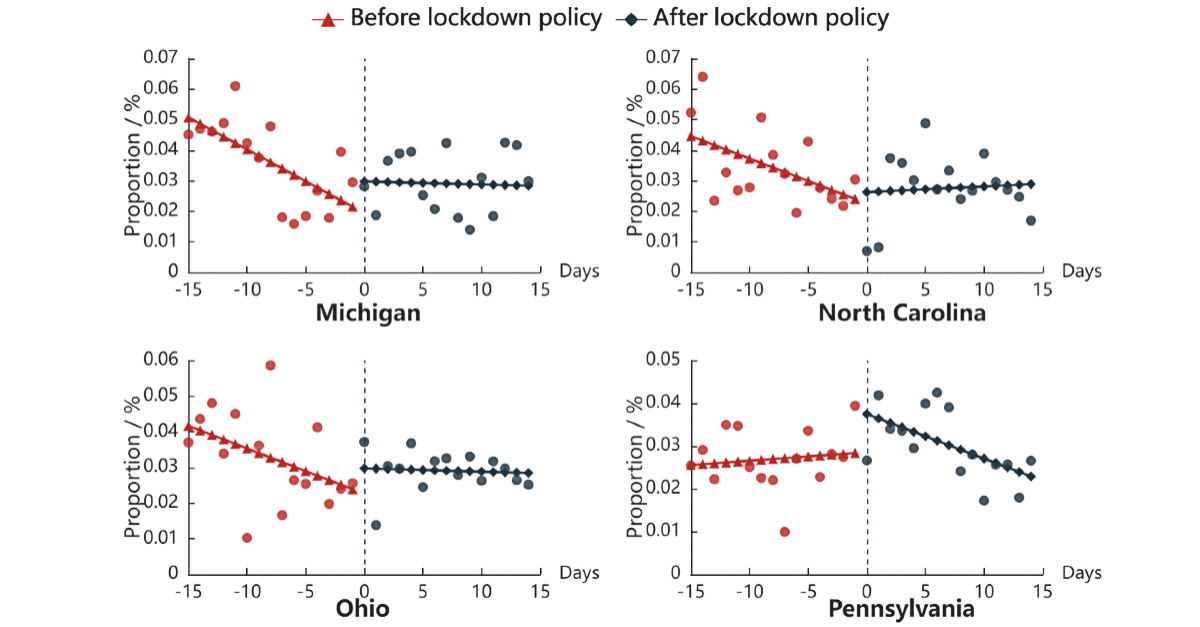
Daily proportion of mental health–related tweets before and after lockdown policies.

We selected 12 states with more than 20,000 related tweets during the study period to explore the effect of lockdown policies on public mental status. We report the significant results found in Michigan, Pennsylvania, North Carolina, and Ohio (analysis results of the other 8 states are displayed in Figure S5 in Multimedia Appendix 1). Sensitivity analysis was applied to verify the stability of the results (Table S4 in Multimedia Appendix 1). Figure 6 shows the proportions of the 4 mental health–related tweets changed after the lockdown policy in Pennsylvania but not in the other 3 states. Table 2 lists the results of the interrupted time series analyses [41] of the lockdown policy on public mental health. The coefficient of “policy,” meaning the change of intercept, was significant in the model of Pennsylvania (*P*=.007), and the coefficient of interaction term indicated that the change of slope was both significant in the models of Michigan (*P*=.03) and Pennsylvania (*P*=.04).

**Table 2:** The impact of lockdown policies on public mental health.

State	Date	Intercept	*P* value	Time^a^	*P* value	Policy^b^	*P* value	Time*policy^c^	*P* value	*F* statistic	*P* value
Michigan	March 24, 2020	0.0528	<.001	–0.0021	.003	–0.0214	.17	0.002	.03	4.669	.009
North Carolina	March 30, 2020	0.0461	<.001	–0.0015	.04	–0.0228	.16	0.0017	.08	2.509	.08
Ohio	March 23, 2020	0.0429	<.001	–0.0013	.03	–0.0117	.39	0.0012	.14	2.078	.13
Pennsylvania	April 1, 2020	0.0254	<.001	0.0002	.63	0.0288	.007	–0.0012	.04	3.033	.046

^a^Time: a continuous variable encoding the number of days in the research period (15 days before and after lockdown).

^b^Policy: a binary variable, encoded as 0 before the lockdown policy and 1 after the policy.

^c^Time*policy: the interaction term of time and policy.

## Discussion

### Principal Findings

We investigated public mental status for 2 and a half years since the beginning of the pandemic by analyzing topics of Twitter discussions, examining potential differences between health care workers and the general population, and studying the impacts of statewide lockdown policies. We found that anxiety and depression problems were frequently mentioned on Twitter during the study period, and the proportion of insomnia discussions increased continuously. The content analysis of mental health–related tweets revealed potential reasons: control measures, economic collapse, pressure from unemployment, and so on. Based on Twitter mentions, we found that all 4 mental health problems studied in this paper (addiction, anxiety, depression, and insomnia) were significantly more prevalent among health care workers than the general population. Finally, lockdown policies had different influences on public mental health status in different states. Among the 12 states studied, the negative effect of lockdown policies on public mental health was significant in Pennsylvania but not the other states.

### Comparison to Prior Works

Consistent with research on similar topics, we found that COVID-19 has severely impacted public mental health and has dynamic influences on public mental health [30,42]. In addition, we found that the proportion of anxiety-related tweets increased to a substantial peak in March 2020 and remained low but stable for several months. A possible explanation is that the outbreak of COVID-19 caused various social problems, such as the shortage of necessities and unemployment, in the initial stage. These problems raised an intense but temporal public fear. As the pandemic continued, public concerns fell to normal as the early-stage issues were mitigated. Another possible explanation is that public emotional response diminishes as the pandemic intensifies, which is consistent with findings from Dyer and Kolic [43]. The remaining 2 peaks of anxiety-related tweets occurred during the presidential election (November 2020) and the fuel price surge (September 2021). The proportion of insomnia also increased during the study period. This observation is consistent with Shi et al [44], who reported an incremental prevalence of insomnia in the follow-up period (from July 8 to August 8, 2020) than the baseline period (from February 28 to March 11, 2020).

The topic analysis shows that the public was concerned about the pandemic, its prevention, and the economic and educational problems caused by COVID-19. Topics such as “social distancing,” “test results,” “world pandemic,” “COVID-19 news,” and “economic collapse” were both observed in our work and previous studies [32,45-49], which only analyzed tweets during the early stage of the pandemic (mainly from January to August 2020). Our study found 2 additional topics through a longer study period: “fuel problems” and “students’ pressure.” These topics correspond to the literature and observations: students (especially children and adolescents) are more vulnerable to psychological disorders [50], and fuel prices frequently fluctuated during COVID-19 [51].

Unlike previous studies that only compare the prevalence of mental health symptoms between health care workers and the general population [52], we also analyzed the topics they focused on. We confirmed that health care workers were more concerned by all the studied mental problems: anxiety, depression, insomnia, and addiction. Particularly, higher proportions of insomnia among health care workers have been extensively reported in the literature [53-57]. These increased problems may be attributed to higher risks of infection [15] and more intense environmental pressure (eg, increased workload, lack of medical supplies, etc) that they face. Health care professionals were more focused on discussing the virus and more interested in sharing news or experiences related to the pandemic, demonstrating a high level of concern about the pandemic, which may be associated with an increased rate of mental disorders.

Lockdown policies had various effects on mental health discussions across US states. In Pennsylvania, it showed a positive association with mental health discussions. However, an opposite association was observed in Michigan, North Carolina, and Ohio. The literature also suggests geographically different associations between local lockdown policies and public mental health. For example, Mittal et al [58] found that most Twitter users shared positive opinions toward lockdown policies in related tweets from March 22 to April 6, 2020, whereas another study focusing on Twitter users in Massachusetts found increased anxiety expression after the enforcement of the Massachusetts State of Emergency and US State of Emergency [59]. Notably, Wang et al [60] found that public sentiment toward lockdown policies was positive in most states (such as Michigan, North Carolina, and Pennsylvania) and negative in only a few states, including Ohio, which also demonstrates geographic variations of public reactions to lockdown policies.

### Strengths and Limitations

Previous work on the same topic has either not focused on the subtypes of mental health problems or studied them over short periods. Our work fills these research gaps by focusing on more granular types of mental health problems over a more extended study period. We built a comprehensive pipeline, including temporal, geographic, and discussion topic analyses; comparisons of trends and topics of concern between groups; and the impact of lockdown policies. On top of the analyses, we released the code and contributed 2 lexicons that can be used to identify mental health issues and health care professionals from tweets.

We also acknowledge the following limitations. First, the evaluation of public mental health on social media is inevitably biased due to the underlying population distribution of social media users. For example, older adults and people with low socioeconomic status may have less access to social media. As a result, this study may not reflect accurate attributes of such subpopulations. However, given the sheer number of people on Twitter, the results of this study are helpful and valuable in tracking public mental health during the pandemic. Additionally, future work could consider sampling according to users’ age to avoid this problem. Second, professional psychologists must make precise diagnoses of mental health problems following official heuristics. Therefore, identifying patients using lexicons based on their tweets can introduce false cases. To validate the reliability of the lexicon, we had professional psychiatrists curate the lexicon based on sampled tweets. Third, tweets that contain keywords do not always reflect the user’s mental health status as they can instead be comments on the news or from other people. To reduce this noise, we removed tweets containing URLs in our preprocessing step, as these tweets were usually summarizations or quotes of different information sources.

### Future Work

The proposed pipeline can be applied to study other public mental health problems, such as suicidal thoughts, posttraumatic stress disorder, paranoia, and so on. It can also be applied to studying characteristics of other cohorts, such as sex minority groups, college students, etc. Regarding the analyses, more data sources (eg, surveys and interviews) could be introduced to validate the conclusions of this research.

### Conclusions

This study developed a comprehensive pipeline to use social media for tracking and analyzing public mental status during a pandemic. It also contributed 2 lexicons that could be used in future studies. We found that the impact of COVID-19 and the corresponding control measures on the public’s mental status is dynamic and shows variability among different cohorts regarding disease types, occupations, and regional groups. Health agencies and policy makers should primarily focus on depression (reported by 51.3% of the tweets) and insomnia (which has had an ever-increasing trend since the beginning of the pandemic), especially among health care workers. Our approach works efficiently, especially when primary studies and large-scale surveys are difficult to conduct. It can be extended to track the mental status of other cohorts (eg, sex minority groups and adolescents) or during different pandemic periods.

## Appendix

Multimedia Appendix 1 Supplementary methods, pictures, and tables.

Multimedia Appendix 2 The proportion and 95% CIs of mental health–related tweets in each state by month.

## Data Availability

The data and code supporting the study’s findings are available at https://github.com/zjumh/mental-health-during-COVID.
