# Supplementary material for: Tracking the Impact of COVID-19 and Lockdown Policies on Public Mental Health Using Social Media: Infoveillance Study
Source: J Med Internet Res. 2022 Oct 13;24(10):e39676. doi: 10.2196/39676 (PMC9566822; doi:10.2196/39676)
Supplement: Multimedia Appendix 1 [file jmir_v24i10e39676_app1.docx]

**Multiple Appendix1**

**Content**

**Methods**

**Figures and Tables**

**Figure S1 A schematic diagram of the study pipeline**

**Figure S2. Trends of four mental health symptom-related tweets**

**Figure S3. Word cloud of mental health-related tweets**

**Figure S4. Perplexity and model coherence of each topic**

**Figure S5 Daily proportion of mental health-related tweets before and after the lockdown policies**

**Table S1. Lexicon for identifying mental health-related tweets**

**Table S2. Lexicon for identifying healthcare workers**

**Table S3. Topics and 20 most probable unigrams/bigrams of each topic**

**Table S4. Sensitive analysis of the lockdown policies (15 days vs. 21 days)**

**References**

**Methods**

**Geographic information extraction**

We concatenated the text of “place” and “location” columns in the tweet metadata, and identified users’ geographic information through keyword matching using USA state names and their abbreviations as listed below:

“Alaska | AK | Alabama | AL | Arkansas | AR | Arizona | AZ | California | CA | Colorado | CO | Connecticut | CT | Delaware | DE | Florida | FL | Georgia | GA | Hawaii | HI | Iowa | IA | Idaho | ID | Illinois | IL | Indiana | IN | Kansas | KS | Kentucky | KY | Louisiana | LA | Massachusetts | MA | Michigan | MI | Minnesota | MN | Missouri | MO | Mississippi | MS | Montana | MT | North Carolina | NC | North Dakota | ND | Nebraska | NE | New Hampshire | NH | New Jersey | NJ | New Mexico | NM | Nevada | NY | New York | NY | Ohio | OH | Oklahoma | OK | Oregon | OR | Pennsylvania | PA | Rhode Island | RL | South Carolina | SC | South Dakota | SD | Tennessee | TN | Texas | TX | Utah | UT | Virginia | VA | Vermont | VT | Washington | WA | Wisconsin | WI | West Virginia | WV | Wyoming | WY”

**Healthcare workers identification**

We used a series of keywords and phrases (**Table S2**) to identify healthcare workers from the self-descriptions of tweet users. To examine the accuracy of this keyword-based identification algorithm, we randomly selected 100 users from the matched users for manual review. We found that 93% of the matched users were healthcare workers. This demonstrates the accuracy of our method.

**Determining the number of topics**

Perplexity evaluates the uncertainty of a document (in our study, a tweet) belonging to a certain topic [39]. Topic coherence evaluates the degree of correlation of words or bigrams in the same topic [37]. A lower perplexity score and a higher topic coherence indicate better model performance. In our case, the optimal number of topics is 16 when comprehensively considering the perplexity, the model coherence, and the topic number (**Figure S1**).

**Interrupted time series analysis**

We chose the proportion of mental health-related tweets as the outcome variable and selected three independent variables to build the linear regression model: 1) time, a continuous variable that encodes the day number in the research period (15 days before and after lockdown); 2) policy, a binary variable encoded as 0 before the lockdown policy and 1 after the policy; 3) the interaction term of time and policy [38]. In regression models, the intercept and coefficient of “time” represent the baseline of the level and slope, respectively; the coefficients of “policy” and interaction term represent the change of level and slope after the lockdown policy. We used the Durbin-Watson test to detect the autocorrelation of residuals. We applied Orthogonal Least Square (OLS) regression model when no autocorrelation existed; otherwise, Generalized Least Square (GLS) model was used.

**Figures and Tables**

**
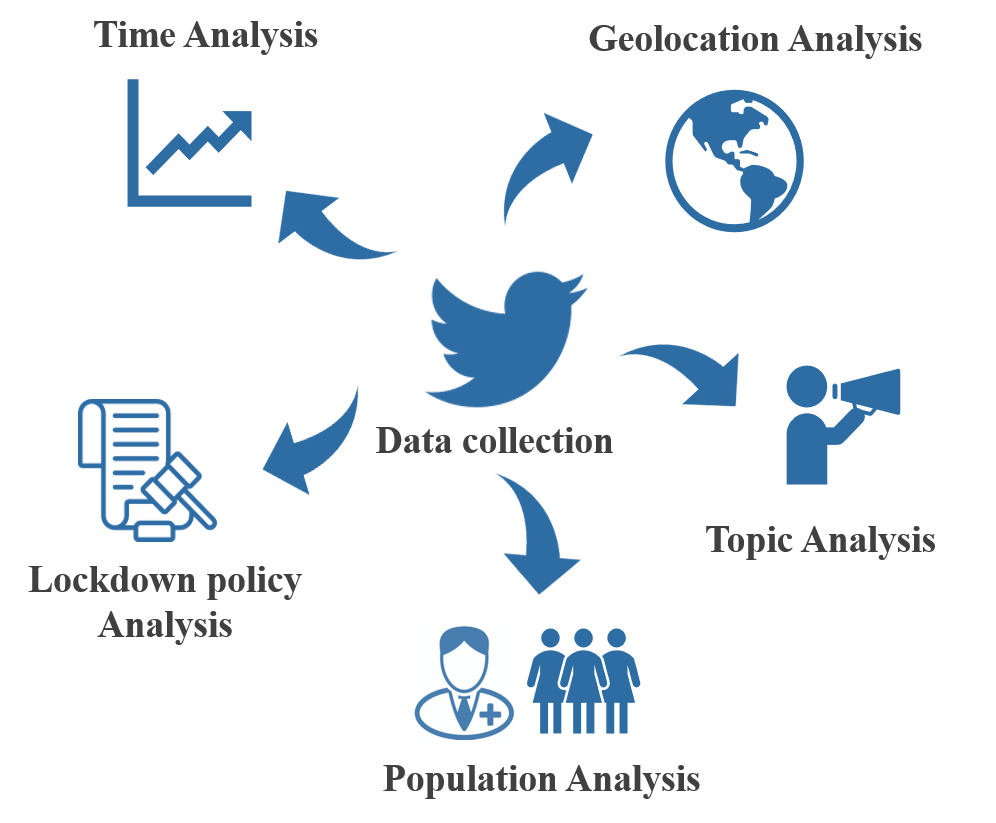
**

**Figure S1 A schematic diagram of the study pipeline**

**
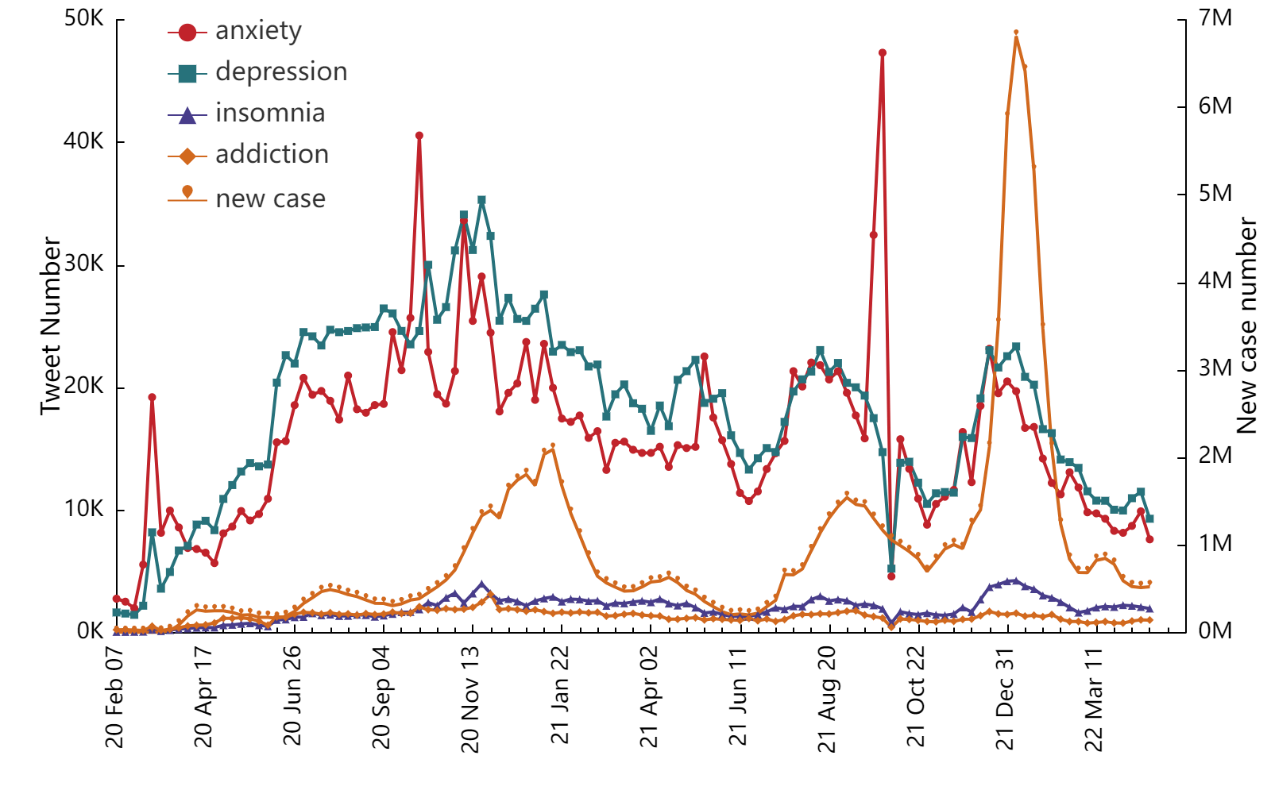
**

**Figure S2. Trends of four mental health symptoms-related tweets**

**
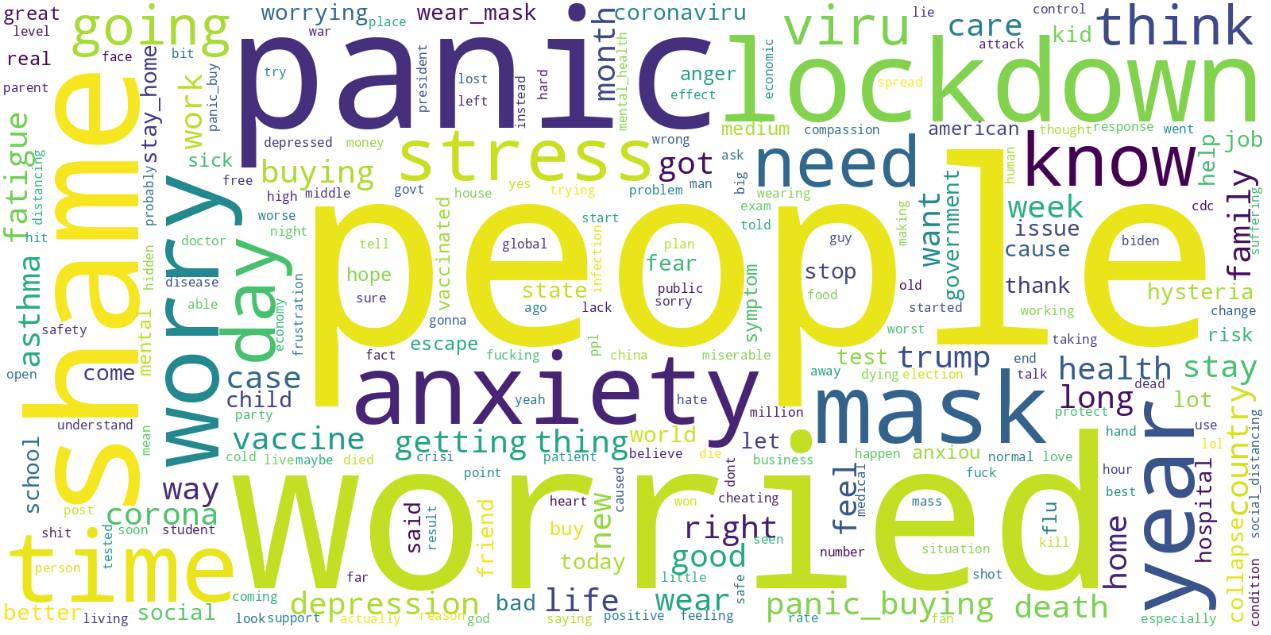
**

**Figure S3.** **Word cloud of mental health-related tweets**

**
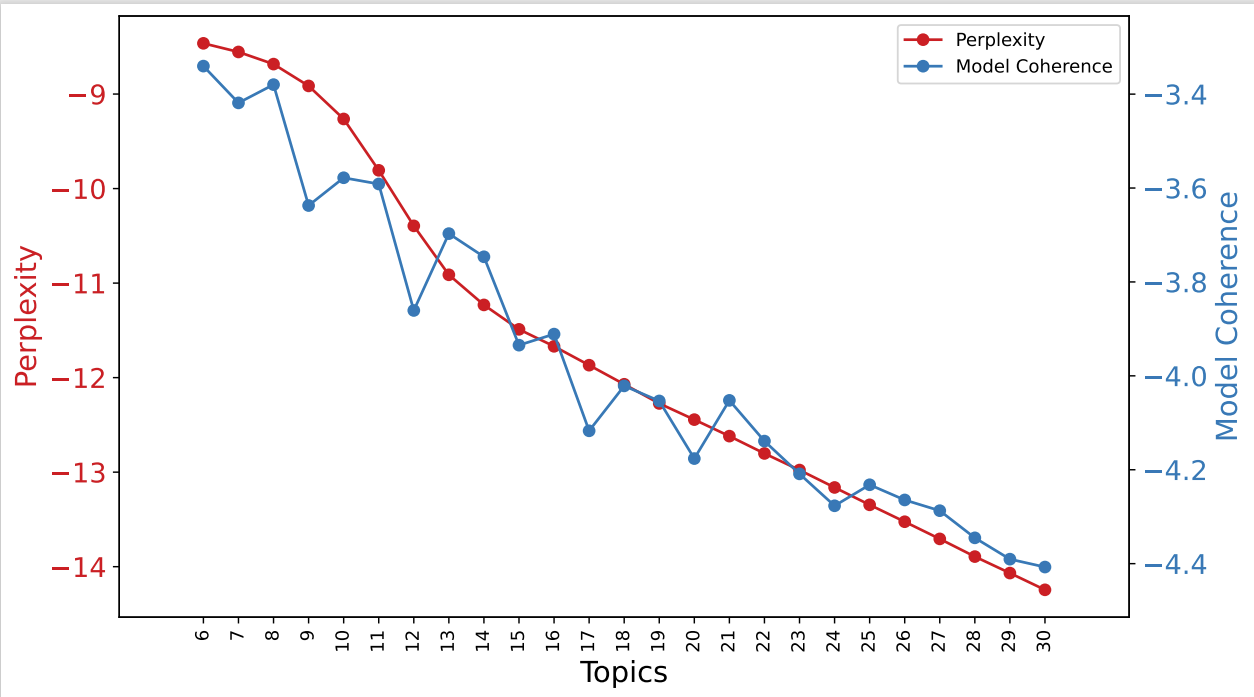
Figure S4. Perplexity and model coherence of each topic**

**
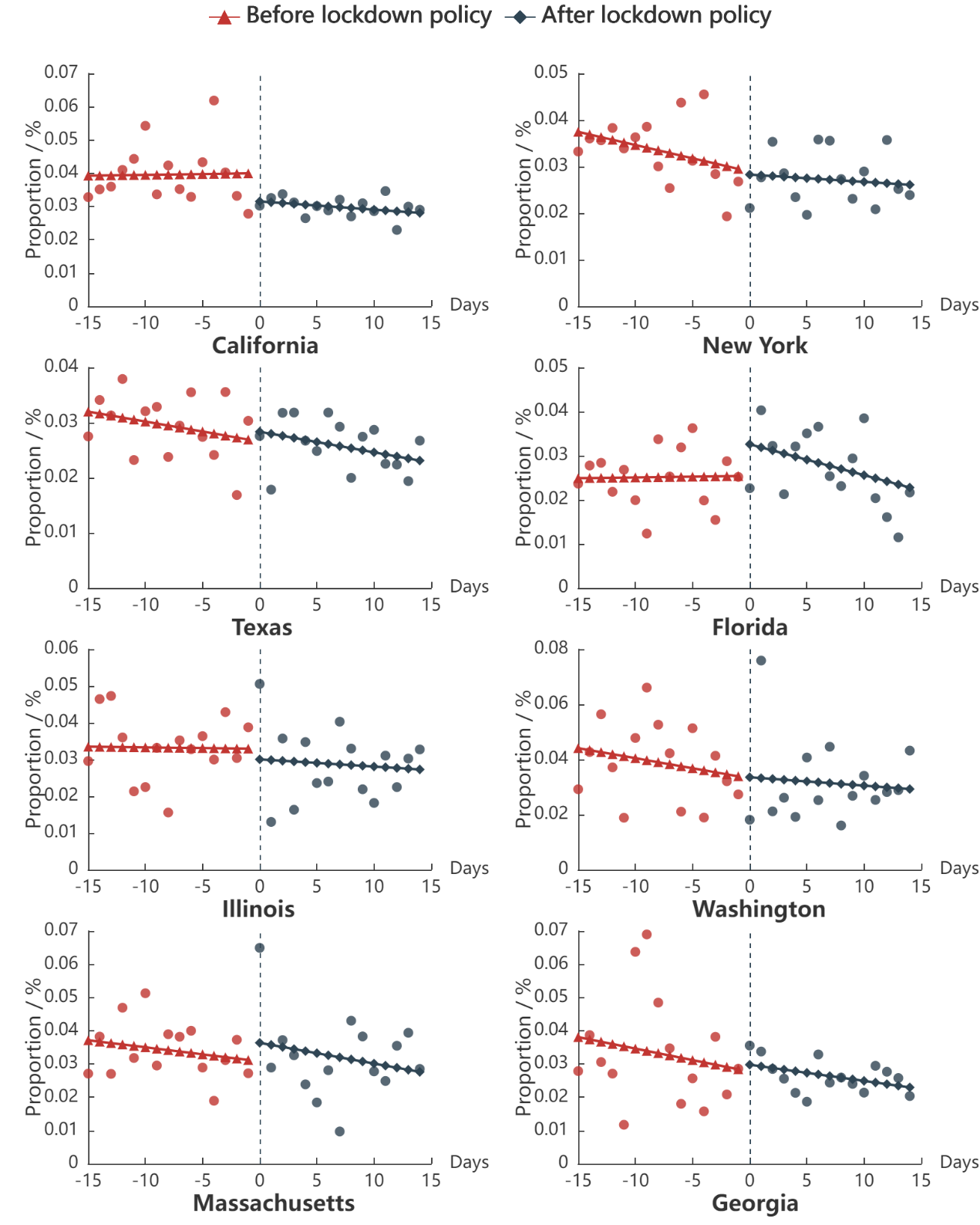
**

**Figure S5. Daily proportion of mental health-related tweets before and after the lockdown policies.** (0 on the X-axis represents the start date of the lockdown policy. The model uses tweets from 15-days before and after the start date.)

**Table S1. Lexicon for identifying mental health-related tweets**

| **Symptoms** | **Keywords** |
| --- | --- |
| anxiety | anxiety \| anxious \| antsy \| restlessness \| concern about the future \| dread fear \| nervousness \| panic \| panic attacks \| paranoia \| worry |
| depression | collapse \| anger \| compassion \| shame \| depressed \| escape \| loneliness \| darkness \| sensitivity \| lupus \| autism \| jealousy \| paranoia \| stress \| postpartum \| hysteria \| asthma \| narcissism \| heroin \| pain mood \| aggression \| immature \| ruthless \| insecurity \| mirage \| cheating \| hidden \| pulse \| projection \| sway \| negative affect \| affect lability \| emotional instability \| emotional stories \| emotional support \| aggressiveness \| snappy \| agitated \| irritable mood \| altered mood \| mood changes \| mood swing \| anger \| frustration \| apathy \| loss of interest \| indifference \| bliss \| elation \| euphoria \| warm fuzziness \| calmness \| depressed mood \| depression \| down feelings \| feeling low \| melancholy \| miserable \| sadness \| feeling empty \| fussiness \| hopelessness \| self-confidence \| stress \| suicidal ideation |
| insomnia | abnormal dreams \| nightmares \| vivid dreams \| asthenia \| disturbed sleep \| insomnia \| sleep problems \| early awakening \| drowsiness \| energy \| lassitude \| listlessness \| exhaustion \| fatigue \| lethargic \| sluggishness \| somnolence \| tiredness \| weariness \| hypersomnia |
| addiction | substance dependence \| physical dependence \| psychological dependence \| harmful use \| withdrawal state \| addiction \| addictive behaviour \| reinforcing \| rewarding \| psychoactive substances \| depressants \| stimulants \| alcohol and drug dependence \| abstinent \| heavy drink \| drinker \| alcoholism \| alcohol intake \| alcohol misuse \| alcohol consumption \| alcohol addiction \| destroyer \| trippy \| alcohol dependence \| alcoholic\| risky drinking \| harmful drinking \| chronic alcohol use \| drinking behavior \| behavior impairment \| chronic alcoholic intoxication \| problem alcohol use \| alcohol-meat \| drinking for pleasure \| avoidance behaviors \| occupational impairment \| marijuana addict \| cocaine addict \| heroin addict \| cannabis addict \| hallucinogen \| opioid abuse \| war on drugs \| illicit use of drugs \| drug affliction \| narcotics abuse \| drug cues \| drug-seeking \| weakening self-regulation \| drug abuse vulnerability \| drug addiction \| prescription drug abuse \| drug habituation \| substance-induced \| drug misuse \| ecstasy \| illicit drug \| chronic drug problem \| drug involvement \| drug reinforcement \| drug relapse \| rehab \| rehabilitation \| nicotine \| harmful smoking \| cigarette smoking \| tobacco consumption \| tobacco chewing \| alternative tobacco product \| dual-tobacco use \| poly-tobacco use \| tobacco dependent \| electric cigarette use \| vape pens \| e-pens \| e-hookah and vape sticks \| smokeless tobacco \| hookah \| cigars \| cigarillos and little cigars \| daily smoking \| tobacco use disorder \| quitting smoking \| internet gaming disorders \| gaming disorder \| internet addiction \| social media addictions \| smartphone addiction \| attention deficit disorder \| problematic online gaming \| disordered gamer \| gaming craving \| video game addiction \| internet game disorder \| maladaptive player game \| problem internet use \| pathological game use \| online game genres \| video-game exposure \| game addiction \| recreational game user \| addictive game behaviors \| gaming community \| gain ability to challenge and dominate others \| acquire status and power \| immersion \| escape real life \| gambling disorder \| pathological gambling \| gambling harm\| problem gambling \| problematic online gambling \| disordered gamblers \| compulsive gambling \| gambling craving \| silent addiction \| problem gambler \| gambling behavior \| preoccupation with gambling \| chronic gambler \| gambling addiction \| gamble harm up \| mounting losses \| gambling-motivated crime \| at-risk gambling \| persistent gambling \| recurrent gambling \| gambling-related cognitive distortion \| uncontrolled gambling \| compulsive gambling \| internet addiction \| problematic use \| problematic phone use \| social media addiction \| addiction |

**Table S2. Lexicon for identifying healthcare workers**

|  | **Keywords** |
| --- | --- |
| **Clinical Occupations** | doctor, physician, surgeon, dentist, ophthalmologist, pediatrician, gynecologist, nurse, Physician, Surgeon, Dentist, Ophthalmologist, Pediatrician, Gynecologist, Nurse |
| **Medical Degrees** | Doctor of Medicine, Bachelor of Nursing, Bachelor of Science in Medicine, Bachelor of Science in Medical Technology, Bachelor of Science in Nursing, Master of Nursing, Master of Science in Medical Technology, Master of Science in Nursing, Doctor of Dental Science, Doctor of Osteopathy, MD, BN, B.S.Med, BSMT, B.S.Med.Tech, BSN, BSNurs, MN, MSMT, MSN, DDS, DO, MBBS, MHS, MPH, RPh, RN, |
| **Title of the Association** | FACP, FACS, FICS, FRCOG, FRCP |

**Table S3. Topics and 20 most probable unigrams/bigrams of each topic**

| Theme | topic | keywords | Explanation |
| --- | --- | --- | --- |
| COVID pandemic | World pandemic | virus, case, china, lockdown, hidden, new, worried, escape, outbreak, number, world, state, country, chinese, worry, worrying, spread, coming, government, data | The COVID pandemic spread around the world. |
|  | Coronavirus | corona, health, virus, coronavirus, mental, protect, ask, safety, mental_health, worried, epidemic, drug, especially, addiction, issue, white, cure, elderly, sound, prevent | People worried about the corona virus and how to cure it, such as drugs. |
|  | Hospital situations | asthma, long, disease, patient, doctor, hospital, risk, heart, condition, lupus, effect, illness, cancer, term, treatment, severe, lung, cough, nurse, immune | In the hospital, doctors, nurses and other medical staffs treated the patients. |
|  | Covid symptoms | day, fatigue, got, week, symptom, hour, post, night, like, second, long, body, today, negative, pain, month, pretty, brain, bad, time | The covid symptoms: fatigue, pain, etc. |
|  | Pandemic life | people, like, think, worry, thing, pandemic, know, life, need, worried, going, want, right, dying, stop, worrying, way, understand, care, lockdown | Public’s life during the pandemic: faced with death. |
|  | Covid-19 news | coronavirus, death, trump, hysteria, people, virus, shame, american, medium, rate, depression, fear, died, million, mass, great, dead, lie, news, kill | The news related to covid deaths from media. |
|  | Test results | worried, flu, vaccine, test, getting, positive, worry, people, tested, vaccinated, testing, got, virus, sick, scared, know, year, case, result, chance | Test results: positive or negative; related symptoms. |
| Preventive measures | Mask wearing | mask, wear, wear_mask, social, distancing, social_distancing, people, wearing, asthma, hand, wearing_mask, worried, cdc, shame, face, public, protest, virus, information, work | Wear mask |
|  | Staying home | shame, home, stay, people, stay_home, pandemic, worried, need, money, job, want, work, care, life, vote, safe, guy, time, leader, party | People stayed home and worried about money and job. |
|  | Lockdown days | pandemic, year, worried, school, time, kid, like, going, anxiety, lockdown, feel, friend, anxious, day, depressed, family, child, work, old, middle | During the lockdown days, people worried about the pandemic with bad emotions. |
| Economic | Panic buying | panic, buying, panic_buying, people, buy, panic_buy, attack, paper, toilet, supply, food, panic_attack, stop, like, medium, store, price, need, shop, toilet_paper | The shortage of supplies resulted in panic buying. |
|  | Fuel problems | dont, worry, decision, car, chronic, major, fuel, governor, police, dont_worry, drive, company, nearly, alcoholic, awful, boy, energy, weight, bitch, date |  |
|  | Economic collapse | pandemic, collapse, economic, response, economy, president, crisis, trump, biden, country, public, war, job, business, security, economic_collapse, financial, nh, government, vulnerable | Economic collapses and crisis. |
| people | Populations | family, shortage, dear, fan, staff, save, appreciate, member, hospital, autism, rehab, patient, worker, team, healthcare, twitter, player, pls, adult, education | Populations: family, staff, patients, healthcare workers. |
| ed^d^ucation | Students’ pressure | student, situation, stress, sir, exam, increase, stock, online, case, corona, pandemic, pressure, calling, help, Chinese_virus, ready, class, administration, college, narcissism | The pandemic influenced school students. |
| Mental health | Mental concerns | anxiety, pandemic, depression, stress, thank, lockdown, time, hard, level, like, global, especially, year, thing, help, going, lot, know, need, think | People felt anxiety, stress, and depression. |

**Table S4. Sensitive analysis of the lockdown policies (15 days vs. 21 days)**

|  | **Date** | **15 days** | | | | **21 days** | | | |
| --- | --- | --- | --- | --- | --- | --- | --- | --- | --- |
|  |  | **Intercept** | **Time^a^** | **Policy^b^** | **Time*Policy^c^** | **Intercept** | **Time^a^** | **Policy^b^** | **Time*Policy^c^** |
| **California** | 2020-03-19 | 0.0392 ^d^ | 4.98e-5 | -0.0037 | -0.0003 | 0.0325^d^ | 0.0005^d^ | 0.0014 | -0.0006 |
| **Florida** | 2020-04-03 | 0.0249 ^d^ | 3.25e-5 | 0.0189 | -0.0007 | 0.0305^d^ | -0.0003 | 0.0110 | -0.0001 |
| **Georgia** | 2020-03-24 | 0.0388 ^d^ | -0.0007 | -0.0012 | 0.0002 | 0.0406^d^ | -0.0005 | -0.0064 | 0.0003 |
| **Illinois** | 2020-03-21 | 0.0335 ^d^ | -3.70e-5 | -0.0003 | -0.0002 | 0.0296 ^d^ | 0.0002 | 0.0050 | -0.0004 |
| **Massachusetts** | 2020-03-24 | 0.0376 ^d^ | -0.0004 | 0.0088 | -0.0002 | 0.0439 ^d^ | -0.0006 | 0.0050 | 0.0000 |
| **Michigan** | 2020-03-24 | 0.0528 ^d^ | -0.0021 ^d^ | -0.0214 | 0.0020 ^d^ | 0.0440 ^d^ | -0.0007 | -0.0093 | 0.0005 |
| **New York** | 2020-03-20 | 0.0380 ^d^ | -0.0006 | -0.0073 | 0.0004 | 0.0332 ^d^ | 0.0000 | -0.0033 | 0.0000 |
| **North Carolina** | 2020-03-30 | 0.046 ^d^ | -0.0015 ^d^ | -0.0228 | 0.0017 | 0.0400 ^d^ | -0.0005 | -0.0065 | 0.0003 |
| **Ohio** | 2020-03-23 | 0.0429 ^d^ | -0.0013 ^d^ | -0.0117 | 0.0012 | 0.0448 ^d^ | -0.0009 ^d^ | -0.0087 | 0.0006 |
| **Pennsylvania** | 2020-04-01 | 0.0254 ^d^ | 0.0002 | 0.0288 ^d^ | -0.0012 ^d^ | 0.0315 ^d^ | -0.0003 | 0.0147 | -0.0003 |
| **Texas** | 2020-04-02 | 0.0324 ^d^ | -0.0004 | 0.0020 | 0.0000 | 0.0351 ^d^ | -0.0004 | -0.0004 | 0.0001 |
| **Washington** | 2020-03-23 | 0.0449 ^d^ | -0.0007 | -0.0064 | 0.0004 | 0.0458 ^d^ | -0.0005 | -0.0020 | 0.0001 |

^a^ Time: a continuous variable encoding the number of days in the research period (15 days before and after lockdown)

^b^ Policy: a binary variable, encoded as 0 before the lockdown policy and 1 after the policy

^c^ Time*Policy, the interaction term of time and policy.

^d^ Statistical significance: *P*<.05
